# Rewiring PTH receptor signaling: Hormone dimerization restores endosomal signaling lost in hypocalcemia-linked PTH mutant

**DOI:** 10.1016/j.jbc.2025.110913

**Published:** 2025-11-05

**Authors:** Jonathan Pacheco, Satyaki Saha, Ji Young Lee, Kelly J. Culhane, Ivet Bahar, Jean-Pierre Vilardaga

**Affiliations:** 1 Department of Pharmacology and Chemical Biology, University of Pittsburgh School of Medicine, Pittsburgh, Pennsylvania, USA; 2 Laufer Center for Physical & Quantitative Biology, Stony Brook, New York, USA; 3 Department of Biochemistry and Cell Biology, Renaissance School of Medicine, Stony Brook University, Stony Brook, New York, USA; 4 Department of Chemistry, Lawrence University, Appleton, Wisconsin, USA; 5 U.S. Department of Veterans Affairs, VA Pittsburgh Healthcare System, Pittsburgh, Pennsylvania, USA

**Keywords:** cAMP, G protein–coupled receptor, parathyroid hormone, PTH receptor, single-molecule biophysics, signal transduction, molecular dynamics, weighted ensemble

## Abstract

G protein–coupled receptors often form dimers and heterodimers at the plasma membrane to transduce signals from various ligands, including peptide hormones. However, the role of homodimerization in regulating signaling by the parathyroid hormone (PTH) type 1 receptor (PTH_1_R) has remained ambiguous. Here, we show that PTH_1_R exists as a monomer in live cells under both basal and ligand-bound conditions, even in the presence of a dimeric form of the PTH mutant, PTH^R25C^ (residue 25 of PTH), which is linked with hypoparathyroidism. Single-molecule fluorescence imaging and single-cell FRET assays support the monomeric behavior of PTH_1_R, with molecular dynamics simulations using weighted ensemble sampling revealing that PTH^R25C^ destabilizes the active conformation of the receptor. In contrast, a synthetic dimeric PTH^R25C^ restores interactions near the receptor’s N-terminal domain, maintains the active conformation, and rescues sustained cAMP signaling. These findings challenge previous assumptions about the homodimerization status of PTH_1_R and highlight how ligand dimerization, rather than receptor dimerization, governs PTH_1_R activation dynamics and location-biased cAMP signaling, offering mechanistic insights relevant to therapeutic strategies against hypoparathyroidism.

The parathyroid hormone (PTH) type 1 receptor (PTH_1_R) is a class B G protein–coupled receptor (GPCR) that regulates mineral ion (Ca^2+^ and PO_4_^3−^) homeostasis as well as bone growth and repair. PTH_1_R signals through the action of its two ligands, PTH and PTH-related protein (PTHrP), by inducing Gs and Gq activation, resulting in cytosolic cAMP and Ca^2+^ elevation, respectively (1). Two modes of cAMP signaling mediated by PTH have been identified. The first is the typical transient cAMP response mediated by activation of the cell surface receptor at the plasma membrane; the second is a sustained cAMP production associated with PTH_1_R endocytosis and its redistribution in early endosomes (2).

The pharmacological significance and disease relevance of the spatiotemporal bias in PTH_1_R signaling *via* cAMP have recently attracted attention. The long-acting PTH (LA-PTH, a modified PTH_1–15_/PTHrP_16–34_ chimera) that induces a prolonged endosomal cAMP production in cells also triggers prolonged production of the active vitamin D, bone formation, and hypercalcemia when injected in mice and monkeys, compared with elevations mediated by PTH_1–34_ (reviewed in Ref. (1)). Conversely, sustained plasma membrane cAMP accumulation induced by the PTH mutant where *l*-Leu7 is epimerized to *d*-Leu7 (PTH^7d^) produced no detectable increase in vitamin D in mice (3). Further studies revealed that a single homozygous Arg-to-Cys mutation in residue 25 of PTH (PTH^R25C^), which causes severe chronic hypocalcemia (low blood Ca^2+^ level) in humans, is defective in inducing endosomal cAMP production in cells and serum Ca^2+^ elevation in mice (4, 5). Collectively, these observations link the duration and location of cAMP signaling to distinct pharmacological and physiological outcomes.

Despite these advances in our understanding of PTH_1_R signaling, we still do not know if the location bias associated with PTH_1_R is regulated by its stoichiometry. Early studies using isolated membranes and chemical crosslinking reported that PTH_1_R is a homodimer in renal cells (6). In addition, studies using only the extracellular domain of PTH_1_R have reported homodimers in basal conditions, dissociating into monomeric form upon PTH binding (7). Studies in cells using a protein-fragment complementation assay (8) or time-resolved FRET (9) suggest that PTH induces the homodimerization of fluorescently tagged PTH_1_R. These apparent discrepancies in PTH_1_R stoichiometry may be partly because of the various systems used, including isolated membranes, truncated receptors, and receptor overexpression. Here, we determined the stoichiometry of the PTH_1_R in basal and agonist-bound states using single-molecule experiments in cells with near-native receptor expression. Furthermore, we carried out molecular dynamics (MD) simulations enhanced by the weighted ensemble (WE) method (10) to study the conformational transitions of the receptor as it switches from active to inactive state as well as FRET photometry and cell signaling experiments. Both computations and experiments showed that the defective signaling and trafficking properties induced by the mutant PTH^R25C^ are rescued by a dimeric form of the hormone stabilized by a disulfide bridge formed between the Cys residues at position 25 of the precursor peptides (referred to as PTH^dimer^). Our study further showed that the PTH^dimer^ acted on the monomeric form of the PTH_1_R.

## Results

### PTH_1_R stoichiometry

**Figure 1:**
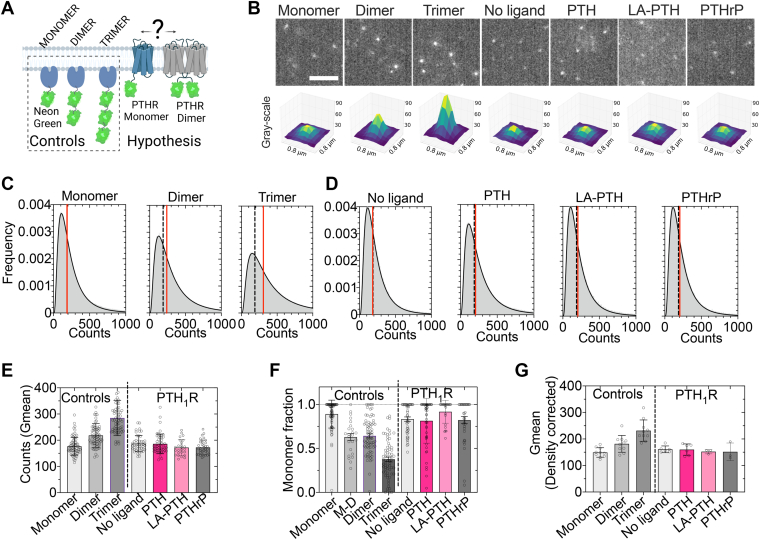
**PTH_1_R stoichiometry.***A*, schematic representation of constructs used to determine PTH_1_R stoichiometry *via* single-molecule analysis in live cells. *B*, *upper panels* show representative images of spots detected in a single frame. Below is the 3D fluorescent profile of an average of 100 random individual spots centered in a square of 0.85 μm by side. The scale bar represents 5 μm. *C* and *D*, frequency of brightness distribution of controls (*C*) and PTH_1_R (*D*). The *vertical red line* represents the geometric mean of the fit obtained with a log-normal function. The *dashed vertical line* representing the geometric mean of the monomer control facilitates comparisons. *E* and *F*, geometric mean of brightness extracted from curves as in *C* and *D*. Each point represents a fit from multiple detections in a single cell (*E*) and monomer fraction from a fit using a sum of two log-normal functions (*F*). Data are the mean ± SD of *n* = 73 (monomer), 24 (mixed monomer/dimer), 75 (dimer), and 68 (trimer) cells for controls and *n* = 47 (no ligand) and 52 (PTH) cells for PTH_1_R. *G*, brightness level corrected by molecular density. The average brightness by cell with its molecular density was fit with a linear regression. The intercept at the *Y*-axis is graphed (trials: monomer, 10; dimer, 10; trimer, 10; PTH_1_R, 6; and PTH_1_R + PTH, 6). Data are the mean ± 95% confidence intervals.

We quantified the stoichiometry of PTH_1_R in the plasma membrane of living human embryonic kidney 293 (HEK293) cells by single-molecule total internal reflection fluorescence (TIRF) microscopy. To this end, we expressed PTH_1_R C-terminally tagged with monomeric NeonGreen (PTH_1_R^mNG^) at a plasma membrane density of ∼0.06 detections/μm^2^, which permits single-molecule imaging as well as signaling as recently demonstrated (11) (Fig. S1). The approach we used involves quantifying molecular brightness from thousands of molecules per cell, measured over 20-s temporal windows. This temporal averaging minimizes artifacts such as photobleaching. In contrast to methods based on photobleaching step analysis, this approach accommodates a certain proportion of incomplete fluorophore labeling per stoichiometric unit since the brightness is averaged across a large number of molecules. Moreover, the photobleaching step analysis typically captures only tens to hundreds of molecules and relies on detecting discrete fluorescence intensity drops; whereas our method benefits from broader sampling, thus enhancing statistical robustness. The average brightness of PTH_1_R^mNG^ molecules was compared with those of monomeric, dimeric, and trimeric controls located at the plasma membrane (Fig. 1*A*). These controls consisted of single or tandem units of mNG fused to the Pleckstrin homology domain of phospholipase Cδ1 (12). To evaluate the sensitivity of this approach, we mixed monomers and dimers in specified ratios (9:1, 8:2, 6:4, and 5:5). Single-molecule brightness increased proportionally with the fraction of dimers, and even a 10% dimer population was significantly different from pure monomers, demonstrating that brightness analysis distinguishes heterogeneous oligomeric states (Fig. S2). We used sparsely distributed molecules of controls and from the PTH_1_R in its resting or ligand-activated state (Fig. 1*B*). A representative 3D fluorescence profile was generated by averaging over 100 random localizations. This profile showed a gradual increase in fluorescence, corresponding to a fluorescence increment in mNG units among the controls (Fig. 1*B*). The number of discrete photobleaching steps also corresponded to the number of fluorescent proteins for the controls (Fig. S3*A*). The PTH_1_R^mNG^ exhibited constant brightness, independently of its agonists, PTH, PTHrP, or LA-PTH (Fig. 1*B*). The average brightness of molecules was determined by fitting the fluorescence intensity to a log-normal distribution (Fig. 1, *C* and *D*). The brightness distributions of PTH_1_R^mNG^ (unliganded and in the presence of different ligands; Fig. 1*D*) and controls (Fig. 1*C*) were compared; the PTH_1_R^mNG^ fluorescence (with/without ligands) was not statistically different from that of the monomer control but significantly different from the dimer and trimer controls (Fig. 1*E* and Table S1). We further evaluated the brightness distribution with a model comprising two brightness populations to verify that PTH_1_R^mNG^ in its basal and agonist-bound conformation was not shifting between monomeric and homodimeric states. Using a two-population model, the PTH_1_R^mNG^ showed no statistical difference from the monomer control (Fig. 1*F* and Table S2). To rule out the presence of mixed monomer and dimer receptor populations, monomeric and dimeric controls were coexpressed in an equimolar ratio. The PTH_1_R^mNG^ exhibited lower brightness with a significantly higher monomer fraction when compared with monomeric and dimeric controls (Fig. 1*F* and Table S2). Given that a higher density of molecules could promote random collisions, potentially producing a higher apparent fluorescence of the spots, we plotted the average brightness as a function of the average number of molecules per cell surface area (molecules/μm^2^). We then used a linear regression fit to account for disparities in molecule density between cells (Fig. S4). After correcting the data by molecular density, the receptor showed no difference from the monomer control (Fig. 1*G* and Table S2). These results are thus inconsistent with PTH_1_R behaving as a dimer in either resting or ligand-bound states.

Next, we investigated whether receptor dimerization can be observed at higher expression levels of the PTH_1_R. We found that overexpression of PTH_1_R C-terminally tagged with either YFP (PTHR^YFP^) or cyan fluorescent protein (PTHR^CFP^) exhibited a FRET efficiency comparable to that of the nonspecific FRET efficiency measured between CFP and YFP molecules localized in the plasma membrane (PM^CFP^ and PM^YFP^, respectively) (Fig. S5, *A*–*C*). These results were further validated using the SPASM (systematic protein affinity strength modulation) (13) approach for the PTH_1_R (see schematics in Fig. S5*D*). We found nonspecific FRET in cells expressing an SPASM PTH_1_R sensor control (PTHR^SPASM^) in the absence or presence of PTH (Fig. S5*D*), but the FRET efficiency was significantly higher in cells expressing the SPASM PTH_1_R for Gq (PTHR^SPASM-Gq^) in the presence of PTH (Fig. S5*D*). These control conditions indicate that our approach can detect specific FRET at the plasma membrane. Although the absence of FRET does not definitively exclude dimerization—since energy transfer efficiency depends on both distance and relative dipole orientation of the fluorophores—prior reports indicate that CFP and YFP retain anisotropy values similar to those measured in aqueous solution when inserted into a GPCR (14). This suggests that their orientation is not substantially perturbed by the receptor environment. Taken together, our findings suggest that the lack of detectable FRET is most consistent with an absence, or very low abundance, of PTH_1_R dimerization under our experimental conditions.

### PTH_1_R stoichiometry with the dimeric PTH

Recent studies have considered the possibility that the functionally defective PTH mutant with Arg-to-Cys mutation at position 25 (PTH^R25C^) found in patients with idiopathic hypoparathyroidism could act as a covalent homodimer because of a potential disulfide bond between C25 residues (15). A synthetic dimeric form of PTH^R25C^ (hereafter referred to as PTH^dimer^) shows calcemic and bone formation responses similar to those of PTH when injected into mice and dogs (15, 16). These observations suggest that the PTH^dimer^ rescues the signaling defective PTH^R25C^, leading us to question the mechanism by which PTH^dimer^ acts on PTH_1_R.

We first tested whether the PTH^dimer^ affected the receptor stoichiometry and compared it with its monomeric counterpart, PTH^R25C^ and PTH. We obtained comparable results in Figure 1, when experiments were repeated on an alternate microscope equipped with a different camera and light source, confirming that the findings were reproducible and not dependent on a specific imaging setup. The tandem controls showed the expected graded fluorescence increment as a function of the number of mNG fluorophores. Individual spots of PTH_1_R showed indistinguishable brightness from the monomer control when bound to PTH^dimer^ or monomeric PTH^R25C^. As a control, PTH_1_R exhibited monomeric brightness in both basal and PTH conditions (Fig. S6, *A*–*C*). A two-population model confirmed that PTH^dimer^ does not modify the monomeric stoichiometry of PTH_1_R (Fig. S6*D*). The approach to correct the variations in molecular density between cells also showed nonsignificant differences of brightness between PTH_1_R and the monomeric control (Fig. S6*E*). These results further confirmed that PTH_1_R is a monomer independently of its basal or ligand-bound state. Moreover, there was no detectable change in the multimerization state of PTH_1_R upon stimulation with PTH^dimer^.

### Interactions of PTH_1_R with different ligands analyzed by MD simulations

**Figure 2:**
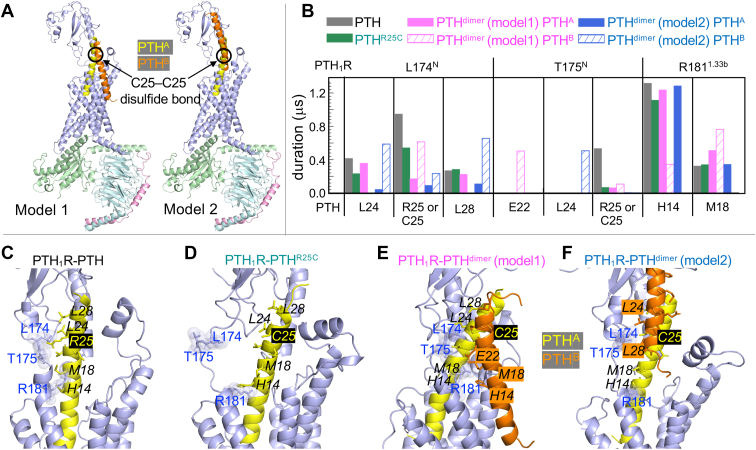
**Molecular dynamics simulations of monomeric and dimeric PTH in PTH_1_R.***A*, two PTH^dimer^-bound PTH_1_R models where a disulfide bond forms between C25 residues of PTH^R25C^ protomers. *B*, interactions (pairs of residues) of L174/T175/R181 with PTH for four systems: PTH_1_R–PTH, PTH_1_R–PTH^R25C^, and the two PTH_1_R–PTH^dimer^. The contact durations for residue pairs are shown in different colors. *C*–*F*, representative snapshots illustrating the interactions of the four systems. Residues for the eight pairs of contacts shown in *B* are displayed in *sticks* and labeled (for dimer models, only the residues contributing to the interactions are labeled for clarity).

Next, we investigated the mechanism by which PTH^dimer^ binds to PTH_1_R using molecular modeling and simulations. The cryo-EM structure resolved for the active state of PTH_1_R bound to Gs (Protein Data Bank code: 6NBF (17)) was used to generate the initial structural models for PTH-, PTH^R25C^-, and PTH^dimer^-bound PTH_1_R. Two alternative structural models were considered for PTH^dimer^ bound to the receptor. In both models, one of the disulfide-bridged PTH protomers of the dimer, designated as PTH^A^, was assumed to adopt the same binding pose as the monomeric PTH; and the second, PTH^B^ was oriented in either parallel/folded state (model 1) or extended state (model 2) (Figs. 2*A* and S7).

Five independent MD simulations of 300 ns (total of 1.5 μs) were conducted for each of the four complexes of PTH_1_R with the ligands (PTH-bound, PTH^R25C^-bound, and two alternative PTH^dimer^-bound forms) in explicit membrane and water. The corresponding RMSD profiles with respect to the initial conformations are shown in panels *A* and *B* of Fig. S8. Panel *C* in the same figure shows the RMSD profile of the ligand PTH^dimer^ in the simulations initiated with model 1 (*left*) and model 2 (*right*). The latter shows enhanced fluctuations because of its fewer number of contacts between the two peptides chains (Fig. S8).

The simulations suggested that selected PTH_1_R N-terminal extracellularly exposed residues (L174^N^ and R181^1.33b^, superscript refers to Wootten’s class B GPCR nomenclature (18)) made persistent interactions with PTH residues, in a ligand-dependent manner. Figure 2*B* summarizes the durations of contacts made by these residues with the specific amino acids (listed along the abscissa) of the ligands. The interactions of L174^N^, T175^N^, and R181^1.33b^ with PTH were observed to be weakened in the complex PTH_1_R–PTH^R25C^, compared with PTH_1_R–PTH, indicating a weaker association of the mutant hormone with the receptor, especially near its mutation site R25C (Fig. 2, *B*–*D*). Notably, these weakened associations were restored or compensated upon PTH^dimer^ binding because of new intermolecular interactions in which PTH^A^ and/or PTH^B^ chains of the dipeptide were engaged. Specifically, strong associations were observed between PTH_1_R residue L174^N^ and PTH^B^ L24 and L28 (in model 2) as well as between R181^1.33b^ and PTH^A^ H14 (models 1 and 2) and M18 (model 1) (Fig. 2, *B*, *E* and *F*). The PTH^dimer^ also exhibited new interactions with T175^N^ at PTH^B^ E22 (model 1) or PTH^B^ L24 (model 2).

Although MD simulations indicate that residues L174^N^ and R181^1.33b^ may help stabilize the interface of the PTH_1_R–PTH^R25C^ dimer, previous experimental results showed that mutating R181^1.33b^ does not affect receptor bioactivity in response to PTH but is necessary for the negative allosteric effect of the small molecule Pitt12 (19). This suggests that, although R181^1.33b^ might not directly influence agonist signaling, it could be involved in regulating conformational dynamics or act redundantly with other residues at the dimer interface. While these findings are hypothesis generating, and future experimental studies (*e.g.*, mutagenesis of L174 or R181^1.33b^) will be needed to test these predictions, the restoration of the weakened PTH^R25C^–PTH_1_R interactions upon replacing PTH^R25C^ by PTH^dimer^ raises the hypothesis that PTH^dimer^ may play a role in rescuing the defective signaling mediated by PTH^R25C^. Notably, the region near R181^1.33b^ has been previously identified as a “hotspot” for regulating location-biased PTH_1_R signaling (1, 20). MD simulations with an enhanced sampling method, WE simulations, were carried out to examine the effects of R25C mutation and dipeptide binding on the activation dynamics of the receptor.

### Effect of PTH^R25C^ mutation and its dimerization on PTH_1_R activation dynamics captured by WE simulations

Toward a mechanistic understanding of the different effects of PTH^R25C^ and PTH^dimer^ binding on the activation dynamics of PTH_1_R, we carried out MD simulations guided by WE methods. WE method permits to generate a broad ensemble of conformations (10). They are particularly useful for examining large changes in protein conformations beyond the range of conventional MD, such as those taking place during activation or deactivation. Here, the WE simulations were performed under the same conditions for three complexes of PTH_1_R, bound to PTH, PTH^R25C^ monomer, and PTH^dimer^ in models 1 and 2, starting from the active conformer of the receptor. In all cases, we examined whether the presence of the mutated or dimerized ligand impacted the conformational dynamics of the receptor, mainly whether the receptor would remain near the initial active state, or tend to reconfigure into its inactive form.

**Figure 3:**
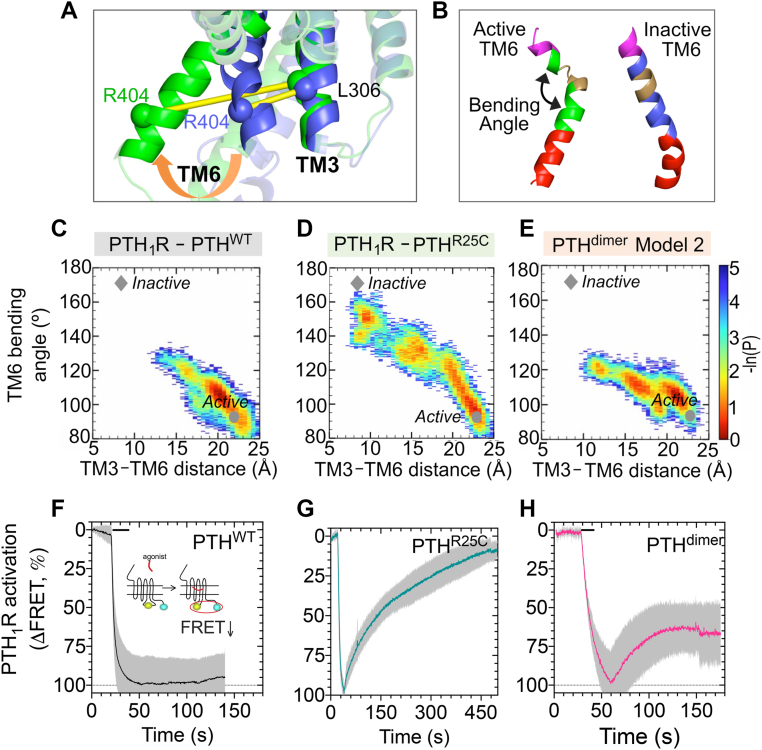
**Progression of the active ligand–bound PTH_1_R conformations toward their inactive states by weighted ensemble (WE) simulations and FRET recordings in live cells.***A* and *B*, the structural features of the TM3–TM6 distance (*A*) and TM6 bending angle (*B*) adopted for visualizing the trajectories of receptor conformations from active toward inactive states. *C*–*E*, WE simulations show the progression of the active to inactive receptor conformations when bound by PTH^WT^ (*C*), PTH^R25C^ (*D*), and PTH^dimer^ (*E*). The PTH^R25C^–PTH_1_R gradually progresses toward its inactive state. In contrast, the PTH^dimer^–PTH_1_R conformations undergo more constrained changes, indicating the active state’s preferential sampling (or retention). -ln(P) represents the free energy. *F*–*H*, the *inset* shows a schematic of the FRET-based PTH_1_R activation sensor (PTH_1_R^CFP/YFP^) with YFP (*yellow*) fused to ICL3 and CFP (*blue*) attached to the receptor C-terminal tail. The graph shows averaged time courses of PTH_1_R activation by recording changes in the FRET ratio in HEK293 cells expressing PTH_1_R^CFP/YFP^. Cells were continuously perfused with control buffer or 1 μM agonist (*horizontal bar*). Means ± SD from N = 10 (PTH), N = 6 (PTH^R25C^), and N = 5 (PTH^dimer^).

The results are presented in Figure 3. To visualize the progression of WE trajectories in each case, we used the conformational space spanned by two coordinates: (1) the PTH_1_R transmembrane (TM) helices TM3–TM6 distance near the cytoplasm, based on residues L306^3.54b^ (TM3, superscript refer to Wootten’s class B GPCR nomenclature) and R404^6.36b^ (TM6) α-carbons (Fig. 3*A*). This distance increases from ∼9 Å (inactive) to ∼22 Å (active) upon activation, and (2) the bending angle of TM6, which varies from ∼93° (active) to 176° (inactive) (Fig. 3*B*). Panels *C*–*E* of Figure 3 display the evolution of receptor conformations (each represented by a dot) sampled by the three complexes, each starting from the active state of the receptor, bound to PTH (Fig. 3*C*), to the mutant PTH^R25C^ (Fig. 3*D*) or to the PTH^dimer^ (Fig. 3*E*, model 2 and Fig. S9, models 1 and 2). Notably, PTH^R25C^-bound PTH_1_R gradually moved to inactive state starting from the active state, as illustrated in Figure 3*D*, indicating that the mutation R25C suppresses the ability of the hormone to stimulate or sustain receptor activation. In contrast, PTH^dimer^-bound PTH_1_R remained in the active or intermediate states, showing limited progression toward the inactive state. Notably, all three cases exhibited some decrease in the TM3–TM6 distance at the cytoplasmic face of the receptor, but the partially bent conformation of TM6 typical of the active state was not maintained in the presence of the mutant hormone PTH^R25C^.

These predictions aligned well with FRET results reporting receptor activation in cells expressing an FRET-based sensor for the PTH_1_R, PTH_1_R^CFP/YFP^ (see schematic in Fig. 3*F*) (21). This sensor reports intramolecular conformational changes in the intracellular loop 3 linking TM6–TM7 that are associated with receptor activation and deactivation through a decrease and increase in FRET, respectively. Ligands were briefly added to HEK293 cells expressing the PTH_1_R sensor, whereas FRET was recorded over time. PTH, PTH^R25C^, and its dimeric form rapidly decreased the FRET ratio, reflecting the stabilization of the active receptor state (Fig. 3, *F*–*H*). Although the active PTH_1_R state switched back to its inactive state with PTH^R25C^ (Fig. 3*G*), it persisted in the case of PTH and PTH^dimer^ (Fig. 3, *F* and *H*). PTH^R25C^ triggered quick receptor activation; however, it reverted to its inactive state during ligand washout. This implies that the ligand probably dissociates from the receptor faster than PTH and PTH^dimer^. There was a strong agreement between WE simulations (Fig. 3, *C*–*E*) and experimental FRET recordings (Fig. 3, *F*–*H*) of PTH_1_R activation, suggesting that PTH^R25C^ destabilizes the active state, driving the transition toward the inactive form of the receptor. In contrast, its dimerization into PTH^dimer^ tends to retain the active state of the receptor, further supporting the view that the dimeric peptide rescues the signaling defects induced by PTH^R25C^.

### PTH_1_R signaling *via* the PTH^dimer^

**Figure 4:**
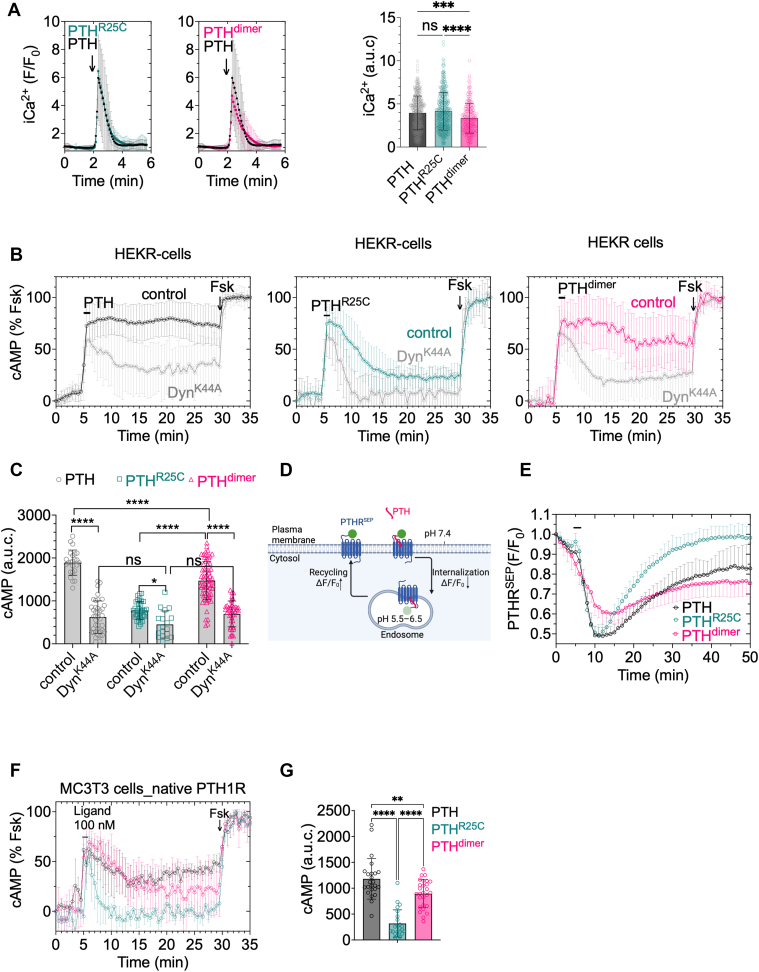
**PTH_1_R signaling.***A*, time courses and integrated responses (area under the curve) of ligand (100 nM) induced intracellular Ca^2+^ elevation in HEK293 cells expressing recombinant PTH_1_R. *Bars* represent the mean values ± SD with n = 200 cells from three independent experiments. *B* and *C*, time courses (*B*) and integrated (*C*) responses of cAMP production after brief stimulation with 1 nM PTH, PTH^R25C^, or PTH^dimer^ in HEK293 cells stably expressing recombinant PTH_1_R without (control) or with dynamin K44A expression (DynK^44A^). The percentage of cAMP responses is relative to the response in the presence of forskolin (FSK). Bars represent the mean values ± SD from n = 24 (PTH), n = 36 (PTH^R25C^), n = 69 (PTH^dimer^) cells in controls and n = 40 (PTH), n = 18 (PTH^R25C^), n = 43 (PTH^dimer^) cells in Dyn^K44A^ from three independent experiments. *D* and *E*, schematic representing the experiment to measure internalization of PTH_1_R^SEP^ (*E*). Time courses showing the internalization profiles for cells stimulated with PTH, PTH^R25C^, and PTH^dimer^. Mean values ± SD with n = 15 cells from three independent experiments (*F*). *F* and *G*, time courses in cAMP production (*F*) and corresponding integrated values (*G*) in MC3T3 cells expressing the native PTH_1_R. Means ± SD from n = 24 (PTH), n = 24 (PTH^R25C^), n = 27 (PTH^dimer^) cells from four independent experiments. Data are the mean ± SD with ∗*p* < 0.05, ∗∗*p* < 0.01, ∗∗∗*p* < 0.001, ∗∗∗∗*p* < 0.0001, and no significant (ns) by one-way ANOVA with Dunnett’s (*A*) and Tukey’s multiple comparison test (*G*).

To test this hypothesis, we measured the time courses of ligand-induced intracellular elevation of cAMP and Ca^2+^ levels, as readouts of PTH_1_R signaling in HEK293 cells stably expressing PTH_1_R. Cells showed the typical transient increase in cytoplasmic Ca^2+^ (iCa^2+^) after exposure to PTH (100 nM). PTH^R25C^ induced a response similar to PTH, whereas PTH^dimer^ induced a slight reduction in iCa^2+^ elevation indicating that Gq coupling was not affected by the mutant and dimer peptides (Fig. 4*A*). For cAMP measurements, cells were continuously perfused with control buffer or peptide ligands. Concentration–response experiments conducted without ligand washout show that PTH, PTH^R25C^, and PTH^dimer^ all induce similar maximum responses and have comparable EC_50_ values (Fig. S10). Time-course recordings confirmed that the addition of 1 nM of ligand increased cAMP accumulation to identical extents (Fig. 4*B*); however, the response induced by PTH^R25C^ returned to its initial level after ligand washout, whereas that induced by its dimeric form or PTH led to prolonged cAMP production (Fig. 4*B*). Given the ability of PTH to induce cAMP production from early endosomes after receptor internalization, we hypothesized that the sustained cAMP effect of PTH^dimer^ might result from its capacity to engage or maintain endosomal PTH_1_R signaling. To test this, we examined the relationship between cAMP duration and receptor internalization. Preventing PTH_1_R internalization by expressing the dynamin K44A mutant significantly affected cAMP mediated by PTH^dimer^ by blocking the sustained cAMP production phase (Fig. 4, *C* and *D*). The inability of PTH^R25C^ to sustain cAMP was partly because of faster recycling of the receptor to the plasma membrane, presumably caused by more rapid ligand dissociation under the mildly acidic conditions found in the endosomes (Fig. 4, *D* and *E*). The rescue of the defective cAMP signaling properties of PTH^R25C^ by its homodimeric form was also observed in osteoblast-derived mouse MC3T3 cells expressing the native PTH_1_R (Fig. 4, *F* and *G*), further validating the observation made on the recombinant cells.

## Discussion

Numerous GPCRs reside as monomers or heterodimers at the plasma membrane (22, 23, 24), and several studies discussed in the *Introduction* section proposed the existence of PTH_1_R dimers in their basal ligand–bound states. Studies indicating the dimerization of other class B GPCRs, such as the secretin receptor (25), further reinforce the idea that the PTH_1_R also dimerizes. Our single-molecule experiments at low receptor expression levels, which still allow for measuring receptor signaling, addressed whether PTH_1_R forms homodimers at the cell surface through a rigorous, quantitative assessment of PTH_1_R stoichiometry. While a transfected, single-cell single-molecule system is a reductionist model, it allows us to isolate receptor-intrinsic behavior with minimal confounding influences. Under these test conditions, we find no evidence that agonist stimulation induces PTH_1_R multimerization. Our data demonstrate that the full-length PTH_1_R is a monomer in its inactive and ligand-activated states in live cells. We found that PTH, PTHrP, and LA-PTH did not alter the receptor stoichiometry. Moreover, the dimeric form of the PTH mutant PTH^R25C^ (encountered in idiopathic hypoparathyroidism), which restores the structural and signaling defects caused by the PTH mutation, also operates through a PTH_1_R monomer. This challenges previous assumptions about the homodimerization status of the PTH_1_R. Our result provides a critical benchmark by showing that ligand-induced dimerization is not an intrinsic feature of PTH_1_R activation under the tested conditions. Determining whether PTH_1_R functions as a monomer or a dimer remains biologically relevant, as stoichiometry can influence activation mechanisms, signaling pathway bias, and opportunities for therapeutic targeting. Using statistically robust and high-resolution methods, our study establishes a necessary reference point in a field where indirect or artifactual claims have often been unchallenged.

MD simulations coupled with the WE method for analyzing the preferential time evolution of conformations as the receptor transitions from active to inactive state provided a molecular understanding of how PTH^R25C^ leads to defective signaling and how it is rescued by its dimeric form. Our earlier studies highlighted the role of the PTH_1_R N-terminal residue R181^1.33b^ and its close neighborhood in allosteric signaling. We discovered nonpeptidic negative allosteric modulators that bind to that particular region (20). This region is understood as a key mechanical site that efficiently propagates conformational changes and, hereby, allosteric signals from the extracellularly exposed PTH-binding site to the intracellular face of the receptor that binds G protein upon activation. The N-terminal domain of the receptor is very flexible (26), and the disruption of the network of contacts around PTH^R25C^ weakens the tight packing and ensuing cooperative response near R181. In the case of the dimeric PTH, however, the occurrence of new contacts with M18, E22, L24, and/or L28 (which were not present in monomeric PTH), in addition to strong interactions at H14, presumably restores the tight packing and cooperativity near the PTH_1_R residue R181 such that one of the PTH protomers in the PTH^dimer^ almost acts as a positive allosteric modulator. Enhanced sampling of the conformational space using WE simulations showed that PTH^R25C^ favors the transition toward the inactive receptor’s state. In contrast, receptor deactivation is resisted in the presence of the dimeric ligand bound to the receptor. The findings, corroborated by FRET experiments that demonstrate the receptor's ability to remain in its active state after binding with PTH and PTH^dimer^, highlight the crucial role of the receptor's N-terminal domain in stabilizing the active form and showcase the utility of WE simulations in accurate sampling of receptor’s conformational space between active and inactive states.

## Experimental procedures

### Cell culture and transfection

Cell culture reagents were obtained from Corning (CellGro). HEK293 (American Type Culture Collection) cells were grown in Dulbecco's modified Eagle's medium (DMEM) (low glucose; Life Technologies, 10567022) supplemented with 5% heat-inactivated fetal bovine serum (FBS; Life Technologies, 10438-034), 100 units/ml penicillin, 100 μg/ml streptomycin (Life Technologies, 15140122) at 37 °C in a humidified atmosphere with 5% CO_2_. Routine mycoplasma testing was performed by using the MycoStrip Mycoplasma Detection Kit from InvivoGen. Testing is performed every 2 months, and cells were free from mycoplasma contamination for all experiments. Receptor expression was controlled using consistent transfection conditions, including low plasmid amounts (200 ng) and short incubation periods post-transfection (∼4 h). This approach alone does not guarantee a specific receptor density; therefore, only cells exhibiting the desired receptor density were selected for single-molecule tracking analyses. The density of receptors was analyzed in all cells and is reported, demonstrating similar densities across all conditions. In addition, we showed that a density of approximately 0.06 molecules/μm^2^ was sufficient to induce a cAMP increase in response to PTH. Mouse osteoblast–like MC3T3-E1 cells, subclone 4 (American Type Culture Collection, CRL-2593-6), were grown in α-MEM supplemented with 10% FBS. All cells were cultured with penicillin (100 units/ml) and streptomycin (100 mg/ml) in a 5% CO_2_ humidified incubator at 37 °C and tested negative for mycoplasma using the MycoStrip Mycoplasma Detection Kit. Signaling studies were performed in HEK293 cells stably expressing the recombinant human HA-tagged PTH_1_R (27). For single-molecule imaging studies, cells were seeded in 35 mm tissue culture dishes with 20 mm number 1.5 cover glass apertures (CellVis) previously treated with 10 μg/ml of human fibronectin protein (Thermo Fisher Scientific, 33016015) for 30 min at 37 °C. Cells were transfected ∼24 h postseeding by using 3 μg Lipofectamine 2000 (Life Technologies, 11668019) in 200 μl Opti-MEM (Life Technologies, 51985091) per dish. Plasmid (0.2 μg) containing PTH_1_R was used to make receptor expression compatible for single-molecule recording. Cells were imaged 4 h post-transfection.

### Chemicals, peptides, and reagents

Human PTH_1–34_ was purchased from Bachem. Recombinant LA-PTH and full-length PTHrP_1–141_ were purified as previously reported (28). Monomeric PTH_1–34_ mutant R25C (PTH^R25C^) and its dimeric form (PTH^dimer^) were chemically synthesized by AnyGen (lot no.: K150993). The purity and mass of each peptide were analyzed by HPLC and MALDI-TOF MS in the manufacturer. Peptides were resuspended in 10 mM acetic acid to make 1 mM stock solution. Forskolin (#344270) was purchased from EMD-Millipore.

### Plasmids

All plasmids were constructed using NEB HiFi assembly or standard DNA recombinant techniques. PTH_1_R was previously described (29). mNG constructs were previously described (30). Human PTH_1_R was cloned into the SPASM sensor construct in a PCDNA5/FRT vector using standard cloning procedures as previously described (13). The SPASM sensor contains PTH1R – 4× GSG – mCitrine – 4× GSG – 10 nm ER/K helix – 4× GSG – mCerulean (FRET donor) – 4× GSG – Gq peptide. The following amino acid sequence was used for the Gq peptide: DTENIRFVFAAVKDTILQLNLKEYNLV. Gly-Ser-Gly (4× GSG) repeats allow for free rotation of each protein domain.

### Single-molecule microscopy

Cells were cultured in 2 ml of prewarmed (37 °C) FluoroBrite DMEM (Life Technologies, A1896702), supplemented with 25 mM Hepes (pH 7.4) and 5% heat-inactivated FBS. Before imaging, cells were rinsed once with 1 ml of FluoroBrite DMEM. Single-molecule imaging was conducted using a Nikon TiE–motorized inverted microscope with a TIRF illuminator and a 100X, 1.45 numerical aperture plan-apochromatic oil-immersion objective. We used two distinct illumination sources, an Oxxius L4C launch equipped with 405, 488, 561, and 638 nm lasers and a Nikon LUN-F laser 405/488/561/640. An ORCA-Fusion BT sCMOS camera (Hamamatsu) (for Fig. 1) and a Prime 95B (Teledyne Photometrics) (for Fig. 2) were used for single-molecule detection. Images were captured with a frame size of 250 × 250 pixels and 1X optical magnification to give a pixel size of 84 or 110 nm for Figures 1 and S5, respectively. Frame exposure was set at 50 ms. Nikon Elements software was used to control the microscope, and image data were saved as ND2 files.

### Brightness analysis

To minimize variations in fluorescence intensity across different experimental days, cells expressing PTH_1_R^mNG^ were excited with fixed laser power and the same region of interest was maintained for all datasets. A 488 nm laser with an exposure of 50 ms per frame was used. Time-lapse recordings were processed with Fiji ThunderSTORM plugin (31) or TrackMate (32). In ThunderSTORM plugin (Fig. 1), the molecular detection settings used 0.22 photons per analog–digital unit. Localizations were determined with a wavelet filter, a local maximum method threshold of 15 to 25 AU, and subpixel localization using an integrated Gaussian point spread function. For TrackMate (Fig. 2), time-lapse recordings were background-subtracted, and the spot detection was obtained using a difference of Gaussians filter with an estimated diameter for detection of 0.5 μm. The total intensity of spots was then used to estimate their brightness. The average photon count was calculated by producing frequency histograms (bin size = 1) of all the spots detected per cell. The resulting distributions were fit with a log-normal function or a sum of two log-normal functions. Note that the fluorescence intensities of single fluorophores imaged by TIRF do not generally obey a normal distribution but instead follow a skewed log-normal distribution, as expected from diffusional motion through the laser beam (33). Because log-normal variables plotted on a linear axis appear skewed, the histogram *x*-axis is shown linearly, whereas the fitting is performed with a log-normal function. On a logarithmic axis, the distribution would appear approximately normal.

The geometric mean was extracted to estimate the central tendency of brightness and compared with concatemers of mNG (mNGx1–3) fused to Pleckstrin homology domain of phospholipase Cδ1 acquired under identical conditions. Histograms and fitting were calculated using custom codes written in Python.

To account for density-dependent effects on molecular brightness, we performed a linear regression of the average molecular brightness (geometric mean, G_mean_) against the fluorescent molecule density (ρ) according to the model: (G_mean_ = ρ·k + G_i_) (Fig. S4), where k is the slope representing density-dependent contributions, and Gi is the *y*-intercept corresponding to the density-corrected intrinsic brightness. For each dataset, contact- and noise-related artifacts were assumed to scale linearly with molecular density within the experimental range. The resulting Gi values were used as the corrected brightness measures for subsequent analysis of oligomerization states (monomer, dimer, and trimer). Each trial was used to obtain enough points to fit a curve. The resulting *y*-axis intercept was graphed per day of trial.

### Models of PTH-, PTH^R25C^-, and PTH^dimer^-bound PTH_1_R for computation

We modeled PTH–PTH_1_R-Gs using the cryo-EM structure resolved for LAPTH–PTH_1_R-Gs (Protein Data Bank code: 6NBF) (17). We mutated LAPTH to PTH, and the missing residues of PTH_1_R (L56–R104, G247–A275, and A394–D398) were constructed using Robetta (34) and ModLoop (35). For PTH^R25C^–PTH_1_R-Gs, we used the same model but with mutation R25C of PTH. For modeling the complexation of PTH^R25C^ dimer with PTH_1_R-Gs, we considered two models as shown in Figure 2*A*. For modeling of PTH^R25C^ dimer, we used the same PTH^R25C^ structure used in PTH_1_R-Gs and aligned the two PTH molecules in parallel in same direction and opposite directions to have the mutation site R25C cysteine form a disulfide bridge as shown in Fig. S6.

### MD simulations

All-atom MD systems with explicit membranes were set up using GHARMM-GUI membrane builder (36), and simulations were performed using NAMD (37) with the CHARMM36m force field (38) for proteins, CHARMM36 lipid (39) for 1-palmitoyl-2-oleoyl-*sn*-glycero-3-phosphocholine, and TIP3P model for water (40). We performed five independent runs of 300 ns each, for four complexes formed by PTH_1_R and different ligands (PTH, PTH^R25C^, and PTH^dimer^, where the protomers are crosslinked in two different conformations). Thus, the individual runs summed up to a total of 6 μs simulations. We relaxed the systems using the equilibration steps in CHARMM-GUI and performed NPT dynamics with a 2-fs time step. Nosé–Hoover constant pressure (1 bar) and temperature (310 K) were used. The same MD setup was used for PTH^R25C^ dimer–containing systems using CHARMM-GUI Solution Builder (41). Contact durations between residues were analyzed using Visual Molecular Dynamics (https://www.ks.uiuc.edu/Research/vmd/), 1.9.4 (42). A contact was defined as any heavy-atom distance <4 Å between a receptor residue and PTH. Contact duration was calculated as the fraction of MD frames in which a given contact was present and then converted to simulation time based on the total number of frames analyzed. Data visualization was performed using the PyMOL Molecular Graphics, version 2.3.5 (Schrödinger, LLC).

### WE simulations

WE simulations were performed using the software WESTPA 2.0 (43). The energy minimized and equilibrated structures of PTH_1_R bound to PTH^R25C^ (monomer), PTH^R25C^ (dimer model 1), or PTH^R25C^ (dimer model 2) were used as the initial conformations for WE simulations. The energy minimization and equilibration steps used AMBER20 (44) with ff14SB (45) force field for the proteins and TIP3P (40) for the solvent. The steps are as follows: (i) 2000 steps of unrestrained minimization: the steepest descent method was used for the first 500 steps, and the next 1500 steps, the conjugate gradient method was used; (ii) 20 ps of restrained NVT equilibration at 298 K using the Langevin thermostat (coupling constant 1/ps) with a 2-fs time step; (iii) 1 ns of restrained NPT equilibration with the same Langevin thermostat and MC barostat; and (iv) 1 ns of unrestrained NPT equilibration using the same Langevin thermostat and MC barostat.

For WE simulations, a one-dimensional progress coordinate was used, which is the distance between TM3 (L306 Cα-atom) and TM6 (R404 Cα-atom). To efficiently sample the rare regions of the energy landscape, the progress coordinate was adaptively divided into bins using the minimal adaptive binning scheme (46) with five trajectories per bin. The minimal adaptive binning scheme was performed with binning in a single direction only, with five evenly spaced bins between leading and lagging trajectories. To evaluate the inactivation tendency of the three systems (Fig. 3, *C*–*E*), 500 iterations of WE simulations were performed in each case, keeping the same sampling direction of active to inactive conformation. The dynamic propagation (50 ps τ) under each iteration was performed using AMBER20 at 298 K, with a 2-fs time step using Langevin thermostat and MC barostat. A 10 Å cutoff was used for nonbonded interactions and particle mesh Ewald method (47) was used for long-range electrostatics. After completion of WE simulations, the trajectories were analyzed using cpptraj (48) and projected onto a two-dimensional space of progress coordinates (Fig. 3, *A* and *B*). The coloring scheme representing the frequency of the sample conformations, *red* being the most frequent.

### FRET recording of receptor activation and deactivation and photobleaching experiments

Recordings were performed using the FRET-based PTH_1_R sensor as previously described (7). HEK293 cells plated on fibronectin-coated glass coverslips were placed at room temperature on Zeiss inverted microscope (Axiovert135) equipped with an oil immersion 100X Plan-Neofluar objective and a photometric system (TILL Photonics). Excitation time was set to 15 ms applied with a frequency of 5 Hz. Individual cells were perfused with buffer containing 150 mM NaCl, 5 mM KCl, 2 mM CaCl_2_, 1 mM MgCl_2_, 20 mM Hepes (pH 7.4), and 0.1% bovine serum albumin without or with ligand for the time indicated by the horizontal bar in graphs. Rapid perfusion was applied using a computer-assisted solenoid valve device (ALA-VM8; ALA Scientific Instruments). Fluorescence was collected by avalanche photodiodes and digitalized with an analog–digital conversion (Digidata1422; Axon Instruments). The emission fluorescence intensities were determined at 535 ± 15 and 480 ± 20 nm (beam splitter dichroic long-pass = 505 nm) upon excitation at 436 ± 10 nm (dichroic long-pass = 460 nm). They were corrected for the spillover of CFP into the 535-nm channel, the spillover of YFP into the 480-nm channel, and the direct YFP excitation to give a corrected FRET emission ratio F^CFP^/F^YFP^. Changes in fluorescence emissions because of photobleaching were systematically subtracted. The change in FRET as a function of time was analyzed using nonlinear regression to a one-exponential model.

FRET between CFP and YFP in cells expressing the receptor constructs was also determined by donor recovery after acceptor bleaching. The emission intensity of CFP was first recorded at 436-nm excitation (CFP^before^), followed by direct illumination of YFP at 500 nm for 3 to 5 min. Subsequently, the emission intensity of CFP was recorded again (CFP^after^). FRET efficiency was calculated according to Equation (1): FRET efficiency = 1 – (CFP^before^/CFP^after^).

### Time course of cAMP and cytosolic Ca^2+^ recordings

Measurements of cAMP production were performed using single-cell FRET-based assays, as described previously (27). In brief, cells were transiently transfected with the Epac1-CFP/YFP cAMP sensor. Then, cells were plated on poly-d-lysine–coated glass coverslips, mounted in Attofluor cell chambers (Life Technologies), and maintained in Hepes buffer containing 150 mM NaCl, 20 mM Hepes, 2.5 mM KCl, 1 mM CaCl_2_, and 0.1% bovine serum albumin, pH 7.4. Imaging was performed on a Nikon Ti-E microscope with a 40X oil-immersion Plan Apo objective (numerical aperture = 1.30) and a motorized stage (Nikon Corporation). For excitation, a mercury lamp was used to stimulate CFP and YFP, with fluorescence emissions captured through 480 ± 20 nm (CFP) and 535 ± 15 nm (YFP) filters. A LUCAS EMCCD camera (Andor Technology) and DualView 2 (Photometrics) with a 505 nm dichroic long-pass filter were used for simultaneous collection. Cells were perfused with either buffer, ligand, or forskolin, as indicated by the horizontal bar in the plots. Fluorescence data were recorded from single cells using Nikon Element Software (Nikon Corporation). The FRET ratio (YFP/CFP) was calculated for each cell, with corrections for background, bleed-through, and photobleaching as described previously (32).

### Statistical analysis

Statistical analyses were processed using GraphPad Prism, version 9 (GraphPad Software). Data were expressed as mean ± SD, 95% confidence intervals, or SSD. Unless indicated, the mean per cell is graphed to produce a grand mean. The data were evaluated using a test for normality and homoscedasticity of variance, respectively, with the D’Agostino–Pearson and Bartlett’s tests. Statistical analyses for data involving one independent variable and three groups were performed using one-way ANOVA with Dunn’s or Dunnett’s multiple comparisons post hoc tests. A *t* test was used for two groups of data. In the single-cell experiments reported here, individual cells (which were captured in the same dish during the same imaging session) are considered biological replicates because of the asynchronous nature of the cell culture. Therefore, each measured cell is considered an independent biological measurement. These measurements were averaged across n cells from N separate experiments.

## Data and material availability

The article and the supporting information contain all the data needed to evaluate the conclusions in the article. Additional data corresponding to single-molecule experiments can be accessed at: https://mynotebook.labarchives.com/share/PTH1R%2520stoichiometry/MTAuNHwxMTY1MTQzLzgvVHJlZU5vZGUvMTc1ODI0MTE4N3wyNi40.

Codes employed for the analysis of single-molecule experiments are available at https://github.com/JPacheco5/Single-molecule-tracking. Materials and source data (stored in Excel 2013 Microsoft Corp) are available upon request.

## Supporting information

This article contains supporting information (Figures S1–S10 and Tables S1–S4).

## Conflict of interest

The authors declare that they have no conflicts of interest with the contents of this article.
