# Supplementary material for: Rewiring PTH receptor signaling: Hormone dimerization restores endosomal signaling lost in hypocalcemia-linked PTH mutant
Source: J Biol Chem. 2025 Nov 5;301(12):110913. doi: 10.1016/j.jbc.2025.110913 (PMC12718132; doi:10.1016/j.jbc.2025.110913)
Supplement: Supporting Figures and Tables [file mmc1.pdf]

Supplementary Information

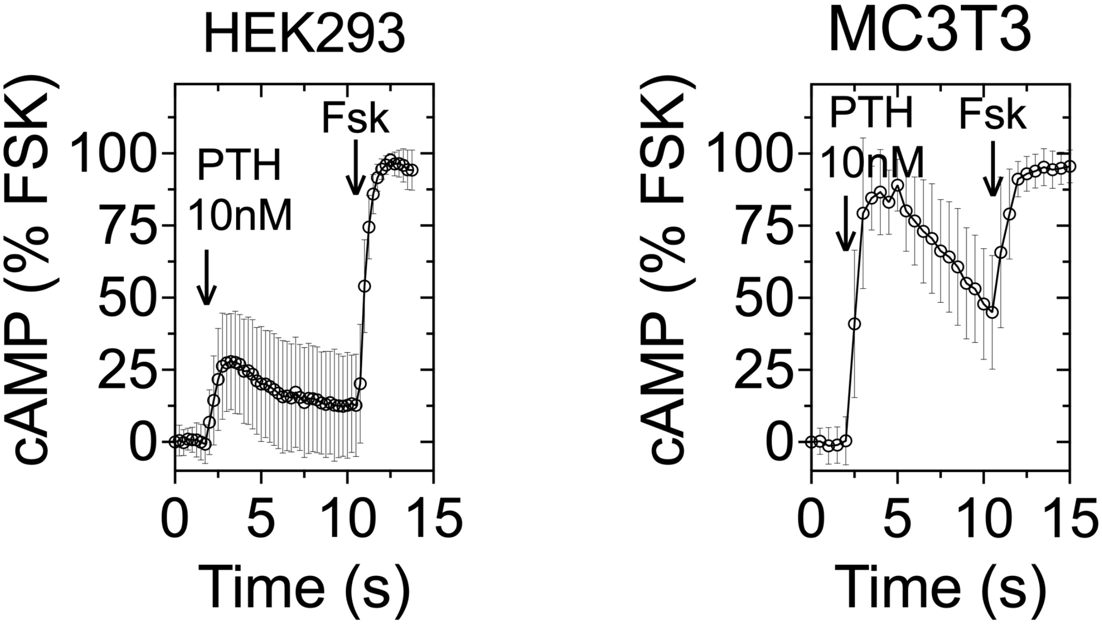

Figure S1. Signaling at a low cell surface of PTH$_1$R density. Time courses of cAMP production in single HEK-293 cells expressing recombinant PTH$_1$R at a cell surface density of 0.06 molecules/μm^2 (*left panel*) and MC3T3 cells (*right panel*) were measured using an intramolecular FRET-based assay. Cells were briefly perfused with 10 nM PTH (horizontal bar). The percentage of the cAMP elevation is relative to the response in the presence of 5 μM forskolin (Fsk). Data are means ± SD of n = 63 (HEK293) and n = 22 (MC3T3) cells *in N* = 3 experiments.

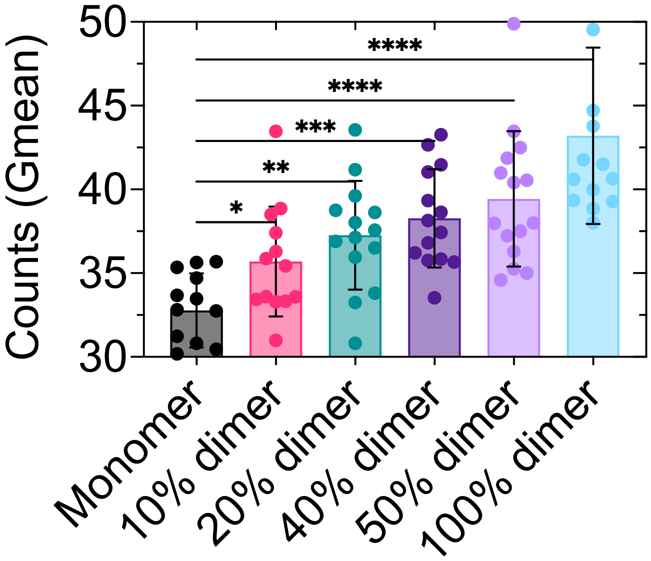

Figure S2. **Brightness analysis on mixed populations of monomers and dimers controls.** Cells were transfected with 200 ng of DNA in total, and the ratios of monomer and dimers plasmids were varied accordingly. The average brightness was calculated from frequency distributions. Data are the mean ± SD with *$P < 0.05$, **$P < 0.01$, ***$P < 0.005$ and ****$P < 0.0001$, by One-way ANOVA with Holm-Šidak comparison test.

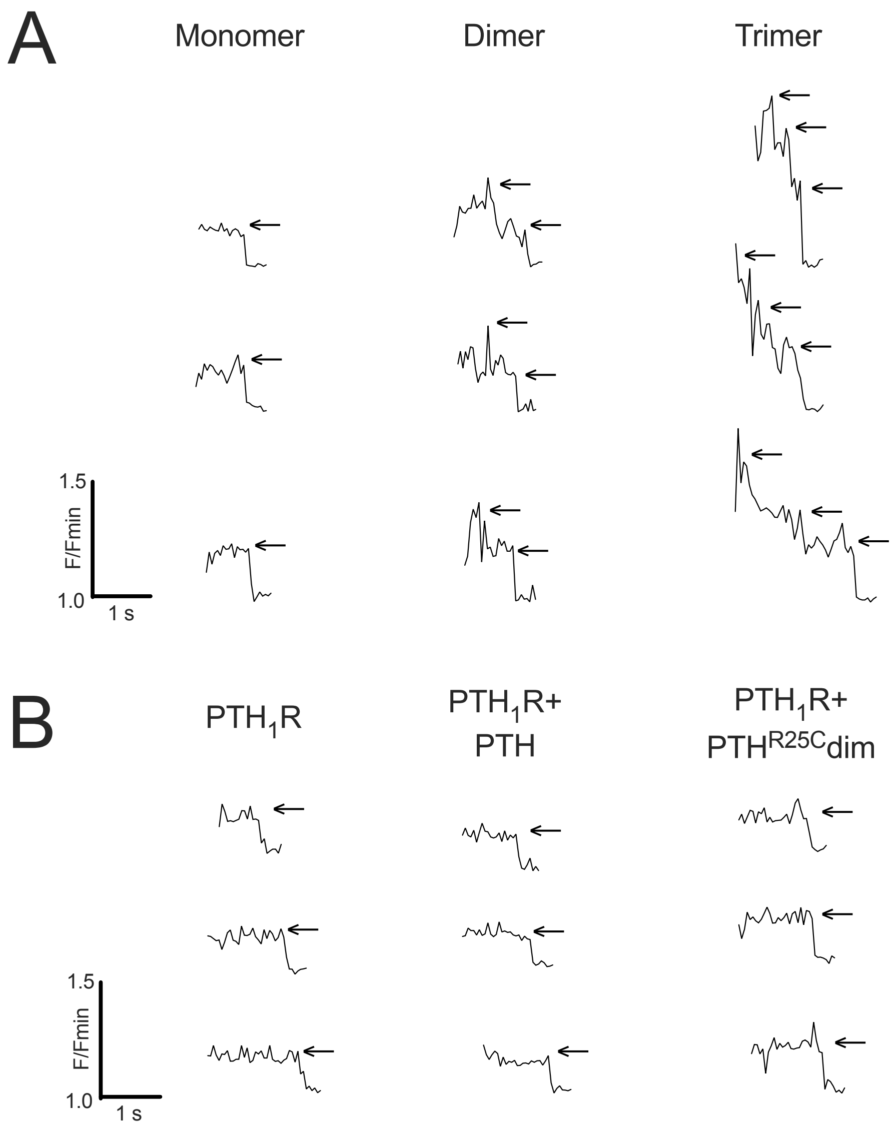

Figure S3. **Representative single-molecule photobleaching steps**. (**A**, **B**) Time courses of fluorescence intensity of single molecules for monomer, dimer, and trimer controls (A), and PTH_1R in basal or activated PTH- and PTH^{dimer}-bound states (B). Each trace corresponds to an individual fluorescent spot. The observation of one, two, or three stepwise reductions in intensity indicates the presence of single, dimeric, or trimeric molecules, respectively.

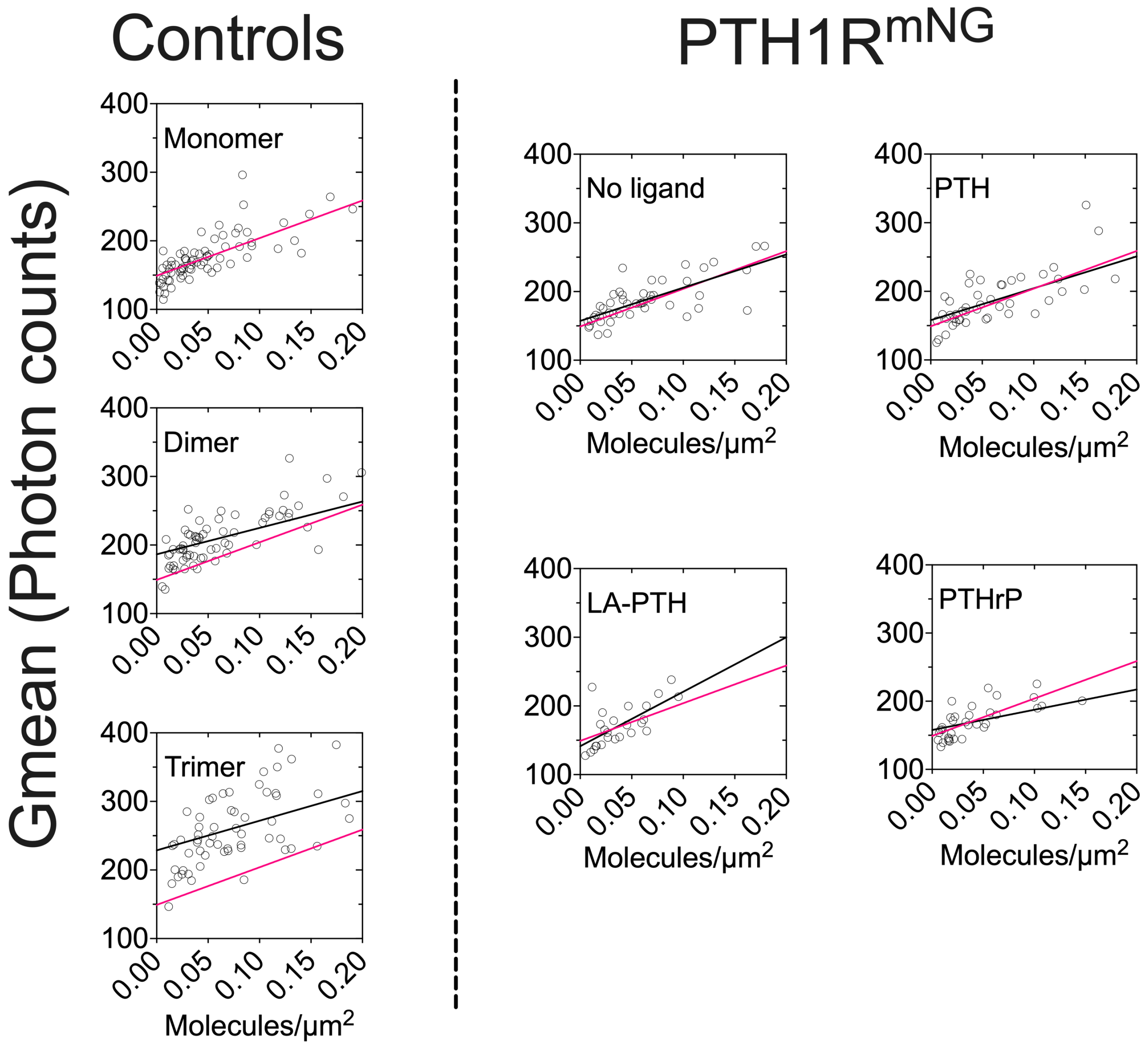

Figure S4. **Corrected brightness analysis**. The average brightness of single molecules per cell (G_{mean}) is plotted against the average number of detections per cell area. The Y-intercept of a linear fit is used to calculate the average brightness corrected by molecular density (as shown in Fig. 1G). For reference, the linear fit of the monomer control is plotted in magenta.

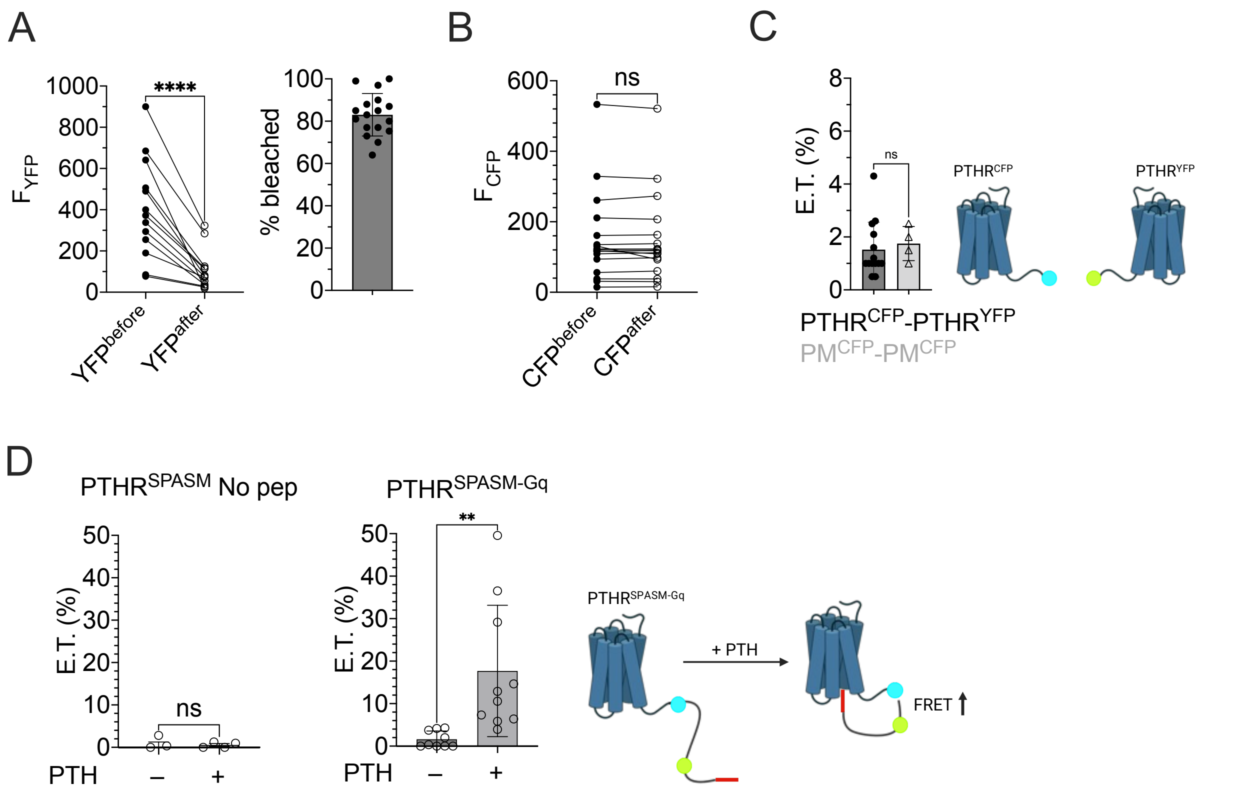

Figure S5. **Testing PTH₁R homodimer at the ensemble level.** (**A–C**) In HEK-293 cells co-expressing PTHR^{CFP} and PTHR^{YFP}, the emission intensities of YFP and CFP were recorded before and after YFP was photobleached for 5 min under light exposure at 500 nm (A, B). FRET efficiency (E.T.) calculated according to equation (1) (C). (**D**) Schematic of the SPASM PTH1R sensors, without and with the Gαq peptide (red), expressed in HEK-293 cells and calculated FRET efficiencies in the absence of presence of PTH (1 µM). Data are the mean ± SD with $^{**}P<0.01$, $^{****}P<0.0001$, and no significant (ns) by t-test.

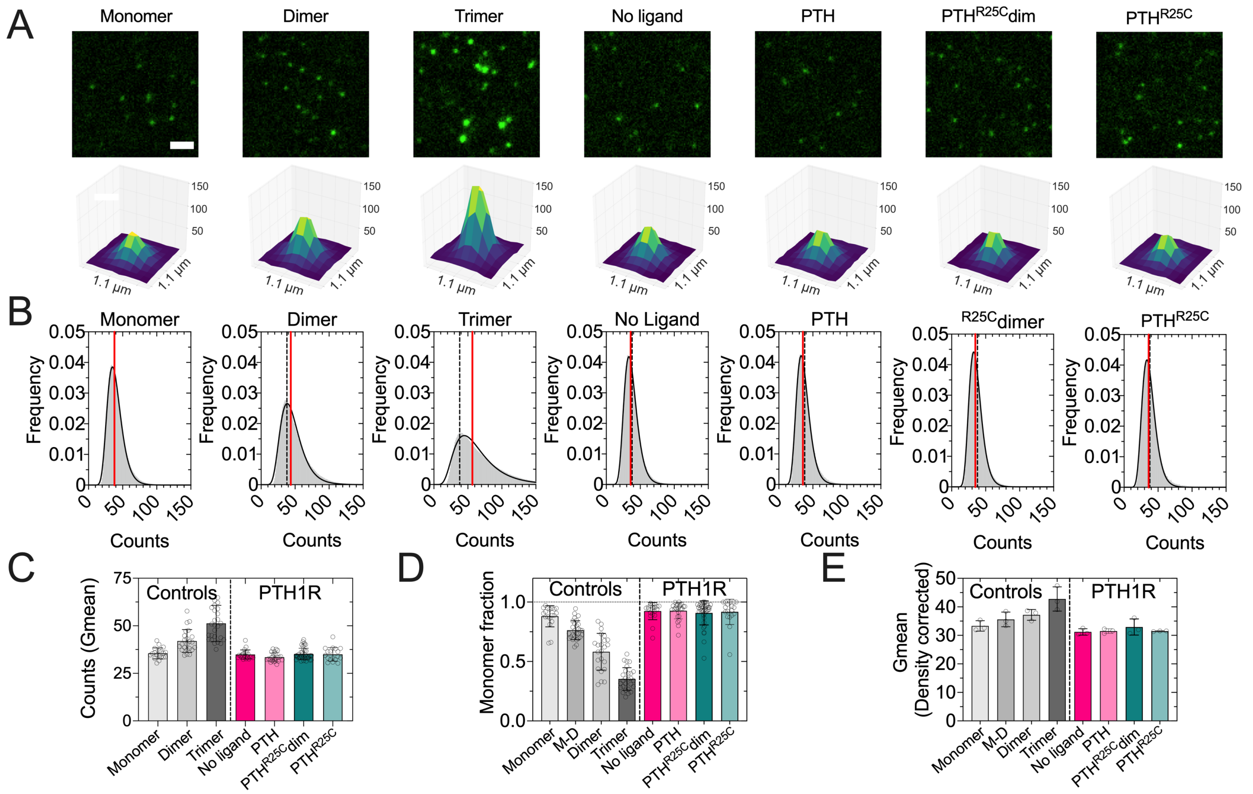

Figure S6. PTH$_1$R stoichiometry with dimeric PTH (A) Upper panels show representative images of spots detected in a single frame. Below is the 3D fluorescent profile of an average of 100 random individual spots centered in a square of 1.1 µm by side. Scale bar, 2 µm.

(B) Frequency of brightness distribution of controls and PTH$_1$R^{mNG}. The vertical red line represents the geometric mean (G_{mean}) of the fit obtained with a log-normal function. The dashed vertical line representing the geometric mean of the monomer control and is shown to facilitate comparisons. (**C, D**) Average brightness calculated as Gmean from frequency distributions as in panel B. Each point represents a fit from multiple detections in a single cell (C). Monomer fraction from a fit using a sum of 2 log-normal functions (D). Data are the mean ± SD of N=19 (monomer), 19 (mixed monomer/dimer), 23 (dimer), and 27 (trimer) cells for controls, and N=20 (no ligand), 24 (PTH), 30 (PTH^{dimer}) and 9 (PTH^{R25C}) cells for PTH$_1$R. (**E**) Brightness level corrected by molecular density. The average brightness by cell in relation by its molecular density was fit with a linear regression. The graph shows the Y-axis intercept from three independent trials conducted on different days for all conditions, except for the PTH^{R25C} condition, where the data is derived from two experimental days. Data are the mean ± SD.

Model 1

Contact duration		
PTH^A	PTH^B	(μs)
M18	M18	1.29
L7	L7	1.09
V21	V21	1.02
H14	H14	0.98
M18	S17	0.97
S17	M18	0.96
Q29	Q29	0.81
M18	V21	0.80
H14	L11	0.79
V21	M18	0.78
L11	H14	0.78
Q29	L28	0.73
N10	L11	0.65
L11	N10	0.61

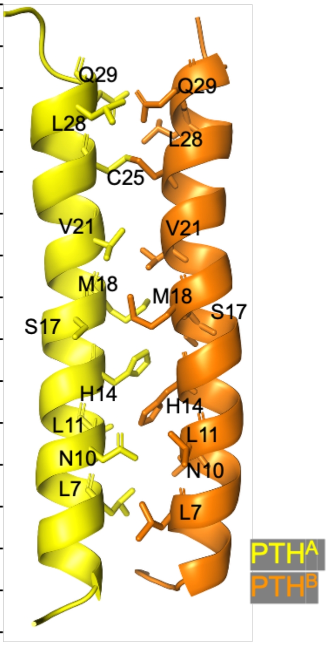

Model 2

Contact duration		
PTH^A	PTH^B	(μs)
Q29	V21	0.94
V21	Q29	0.90
L28	V21	0.83
L28	L24	0.83
V21	L28	0.77
L24	L28	0.74
C25	V21	0.74
M18	Q29	0.70
V21	C25	0.64

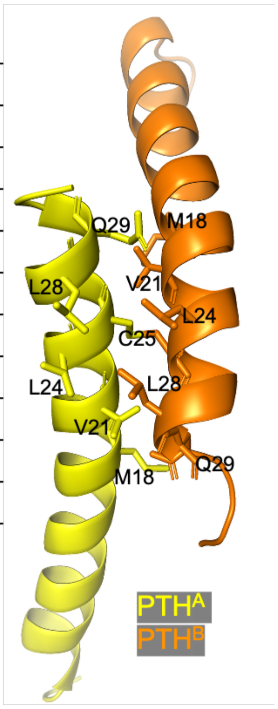

Figure S7. Representative PTH^{dimer} configurations and inter-peptide contact durations observed during MD simulations of their complexes with PTH$_1$R. Contact durations of pairwise interactions between PTH protomers disulfide-linked via C25 in the PTH^{dimer} based on 5 runs of 300 ns each (a total of 1.5 μs) are listed for Model 1 (left) and Model 2 (right) used in two sets of simulations.

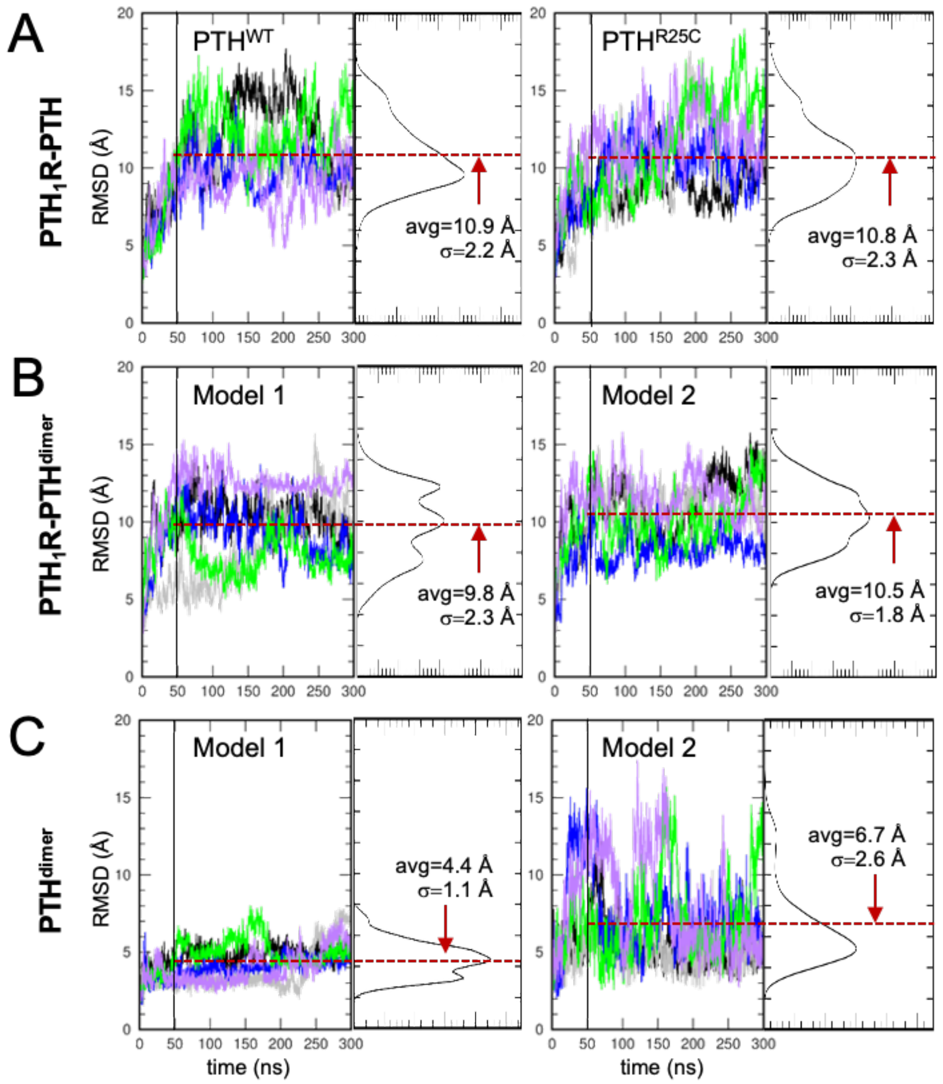

Figure S8. Time evolution of RMSD profiles for the complexes of PTH$_1$R with different ligands. Results from five runs of 300 ns each are shown in each case in different colors, and the corresponding cumulative histograms based on the time interval 50 ~ 300 ns are shown along the right ordinates. Their average values (*red dashed line*) and standard deviations are reported. Transmembrane domains of PTH$_1$R in different snapshots were aligned before calculating the RMSDs. Results are presented for **(A)** the two complexes formed between PTH$_1$R and PTH (*left*) or PTH^{R25C} (*right*); **(B)** the two PTH^{dimer}-bound PTH$_1$R models with disulfide bonds between R25C residues of PTH^A and PTH^B protomers; and **(C)** Two PTH^{dimer} models.

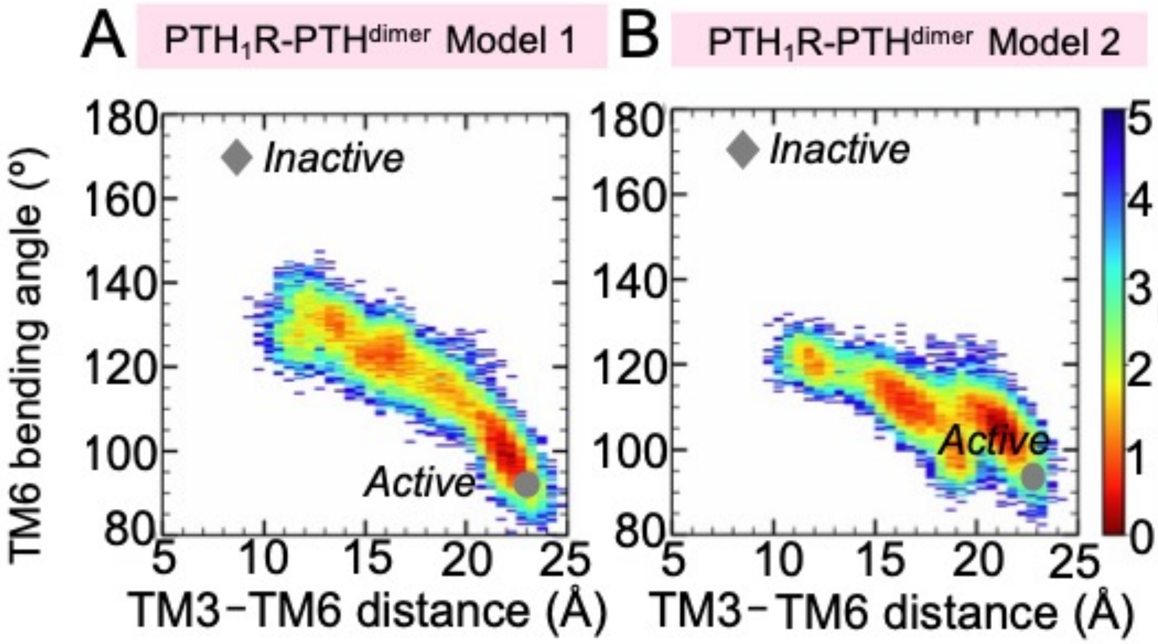

Figure S9. Progression of the active PTH^{dimer}-bound PTH$_1$R conformations towards their inactive states during weight ensemble (WE) simulations. (A, B) WE simulations show that PTH$_1$R bound to PTH^{dimer} preferentially samples conformations near the active state, and progresses toward an intermediate state, but not the inactive state. The same behavior is observed for both Models 1 and 2, shown in Fig. 2A.

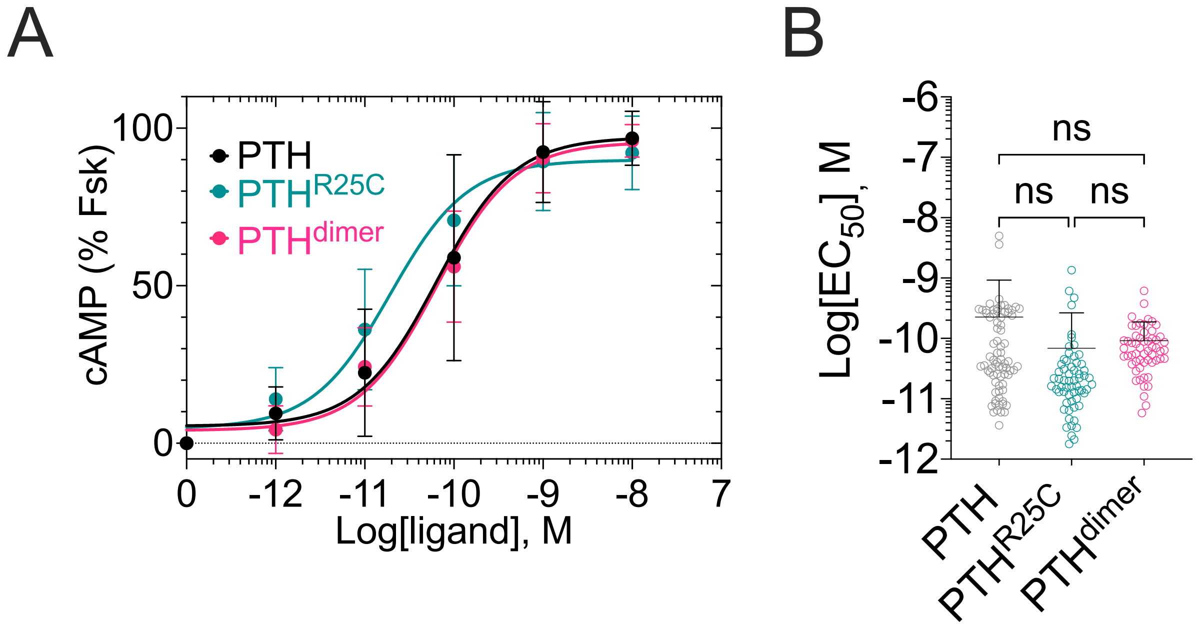

Figure S10. Concentration-response curves. (A) cAMP peak production in response to increasing ligand concentrations in HEK293 cells stably expressing PTH_1R and the cADD-green-down cAMP sensor. The percentage of cAMP responses is relative to the response in the presence of forskolin (Fsk). (**B**) Mean values ± SD of EC_{50} values from n = 75 (PTH), n = 58 (PTH^{R25C}), and n =61 (PTH^{dimer}) cells in N = 3 experiments. No significant (ns) statistical differences by one-way ANOVA.

Table S1. *P* values of average brightness data in Figure 1E by One-way ANOVA with Krustal-Wallis and Dunn's multiple comparison test. Significant results are highlighted in bold.

**Constructs**	**Monomer**	**Dimer**	**Trimer**	**PTH$_1$R**	**PTH$_1$R+ PTH**	**PTH$_1$R+ LAPTH**	**PTH$_1$R+ PTHrP**
**Monomer**		**<0.0001**	**<0.0001**	>0.9999	>0.9999	>0.9999	>0.9999
**Dimer**	**<0.0001**		**<0.0001**	**0.0101**	**0.0011**	**0.0001**	**<0.0001**
**Trimer**	**<0.0001**	**<0.0001**		**<0.0001**	**<0.0001**	**<0.0001**	**<0.0001**
**PTH$_1$R**	>0.9999	**0.0101**	**<0.0001**		>0.9999	>0.9999	>0.9999
**PTH$_1$R+PTH**	>0.9999	**0.0011**	**<0.0001**	>0.9999		>0.9999	>0.9999
**PTH$_1$R+LAPTH**	>0.9999	**0.0001**	**<0.0001**	>0.9999	>0.9999		>0.9999
**PTH$_1$R+PTHrP**	>0.9999	**0.0001**	**<0.0001**	>0.9999	>0.9999	>0.9999	

Table S2. *P* values of monomer fraction data in Figure 1F by One-way ANOVA with Krustal-Wallis and Dunn's multiple comparison test. Significant results are highlighted in bold.

**Constructs**	**Monomer**	**M-D**	**Dimer**	**Trimer**	**PTH$_1$R**	**PTH$_1$R+ PTH**	**PTH$_1$R+ LAPTH**	**PTH$_1$R+ PTHrP**
**Monomer**		**<0.0001**	**<0.0001**	**<0.0001**	>0.9999	>0.9999	>0.9999	>0.9999
**M-D**	**<0.0001**		>0.9999	0.1386	**0.0180**	**0.0237**	**0.0002**	**0.0161**
**Dimer**	**<0.0001**	>0.9999		**0.0002**	**0.0006**	**0.0008**	**<0.0001**	**0.0010**
**Trimer**	**<0.0001**	0.1386	**0.0002**		**<0.0001**	**<0.0001**	**<0.0001**	**<0.0001**
**PTH$_1$R**	>0.9999	**0.0180**	**0.0006**	**<0.0001**		>0.9999	>0.9999	>0.9999
**PTH$_1$R+PTH**	>0.9999	**0.0237**	**0.0008**	**<0.0001**	>0.9999		>0.9999	>0.9999
**PTH$_1$R+LAPTH**	>0.9999	**0.0002**	**<0.0001**	**<0.0001**	>0.9999	>0.9999		>0.9999
**PTH$_1$R+PTHrP**	>0.9999	**0.0161**	**0.0010**	**<0.0001**	>0.9999	>0.9999	>0.9999	

Table S3. *P* values of average brightness data in Figure S5C by One-way ANOVA with Krustal-Wallis and Dunn's multiple comparison test. Significant results are highlighted in bold.

**Constructs**	**Monomer**	**Dimer**	**Trimer**	**PTH$_1$R**	**PTH$_1$R+ PTH**	**PTH$_1$R+ ^{R25C}dimer**	**PTH$_1$R+ PTH^{R25C}**
**Monomer**		**0.0229**	**<0.0001**	>0.9999	>0.9999	>0.9999	>0.9999
**Dimer**	**0.0229**		>0.9999	**0.0030**	**<0.0001**	**0.0002**	**0.0002**
**Trimer**	**<0.0001**	>0.9999		**<0.0001**	**<0.0001**	**<0.0001**	**<0.0001**
**PTH$_1$R**	>0.9999	**0.0030**	**<0.0001**		>0.9999	>0.9999	>0.9999
**PTH$_1$R+PTH**	>0.9999	**<0.0001**	**<0.0001**	>0.9999		>0.9999	>0.9999
**PTH$_1$R+^{R25C}dimer**	>0.9999	**0.0002**	**<0.0001**	>0.9999	>0.9999		>0.9999
**PTH$_1$R+PTH^{R25C}**	>0.9999	**0.0002**	**<0.0001**	>0.9999	>0.9999	>0.9999	

Table S4. *P* values of average brightness data in Figure S5D by One-way ANOVA with Krustal-Wallis and Dunn's multiple comparison test. Significant results are highlighted in bold.

**Constructs**	**Monomer**	**M-D**	**Dimer**	**Trimer**	**PTH$_1$R**	**PTH$_1$R+ PTH**	**PTH$_1$R+ ^{R25C}dimer**	**PTH$_1$R+ PTH^{R25C}**
**Monomer**		0.5311	**0.0020**	**<0.0001**	>0.9999	>0.9999	>0.9999	>0.9999
**M-D**	0.5311		>0.9999	**0.0056**	**0.0023**	**0.0007**	**0.0004**	**0.0031**
**Dimer**	**0.0020**	>0.9999		>0.9999	**<0.0001**	**<0.0001**	**<0.0001**	**<0.0001**
**Trimer**	**<0.0001**	**0.0056**	>0.9999		**<0.0001**	**<0.0001**	**<0.0001**	**<0.0001**
**PTH$_1$R**	>0.9999	**0.0023**	**<0.0001**	**<0.0001**		>0.9999	>0.9999	>0.9999
**PTH$_1$R+ PTH**	>0.9999	**0.0007**	**<0.0001**	**<0.0001**	>0.9999		>0.9999	>0.9999
**PTH$_1$R+ ^{R25C}dimer**	>0.9999	**0.0004**	**<0.0001**	**<0.0001**	0.9993	>0.9999		>0.9999
**PTH$_1$R+ PTH^{R25C}**	>0.9999	**0.0031**	**<0.0001**	**<0.0001**	>0.9999	>0.9999	>0.9999	
